# Molecular Analysis of *Plasmodium ovale* Variants

**DOI:** 10.3201/eid1007.030411

**Published:** 2004-07

**Authors:** Thin Thida Win, Amadu Jalloh, Indah Setyawati Tantular, Takafumi Tsuboi, Marcelo Urbano Ferreira, Masatsugu Kimura, Fumihiko Kawamoto

**Affiliations:** * Nagoya University Graduate School of Medicine, Nagoya, Japan;; † Airlangga University, Surabaya, Indonesia;; ‡ Ehime University, Matsuyama, Ehime, Japan;; § University of São Paulo, São Paulo, Brazil;; ¶ Osaka City University Medical School, Osaka, Japan

**Keywords:** Plasmodium ovale, variant isolate, classic isolate, molecular analysis, SSUrRNA gene, cysteine protease gene, ookinete surface protein gene, cytochrome b gene, Southeast Asian isolate

## Abstract

Complete DNA sequences of the small subunit ribosomal RNA (*SSUrRNA*) gene and partial sequences of three other loci were obtained from three variant-type and three classic-type *Plasmodium ovale* isolates from Southeast Asia and compared with GenBank-available data. Three different *SSUrRNA* sequences (Pov 1–3) were found in each variant-type isolate, and two different *SSUrRNA* sequences (Poc 1–2) in each classic-type isolate. Pov 1–3 were closer to sequences previously found in the Cameroon and MAL/MAI isolates, whereas Poc 1–2 were closer to sequences previously found in two clones of the Nigerian I/CDC strain. The 3´ half of Pov 1–3 was identical to the partial sequence of the *SSUrRNA* gene from the London School (LS) strain. Results support grouping *P. ovale* into two groups, the classic type (including the Nigerian I/CDC strain) and the variant type (Cameroon, MAL/MAI, and LS isolates).

## Abstract 2

Sequence analyses of six isolates from Southeast Asia supports dividing species into types

The geographic range of the human malaria parasite *Plasmodium ovale* has been thought to be mostly limited to tropical Africa, the Middle East, Papua New Guinea, and Irian Jaya in Indonesia; it has rarely been described in other countries of Southeast Asia. More recently, however, with the aid of polymerase chain reaction (PCR)-based species identification and improved microscopic techniques, *P. ovale* infections have been frequently reported in Southeast Asia (1,2). *P. ovale* may represent an emerging cause of benign and relapsing tertian malaria in this region or, alternatively, may have been overlooked in previous surveys based on classic microscopy techniques (1). The widespread distribution of *P. ovale* in Southeast Asia affects the choice of appropriate drugs for malaria chemoprophylaxis in travelers, since most currently used regimens are not effective against the dormant liver stages of *P. ovale* and *P. vivax*, which may cause relapses several months after the primary infection (3).

During our previous molecular studies of *P. ovale* in southern Vietnam (4), we found two field isolates whose partial sequences at the block 9 region (5) of the small subunit ribosomal RNA (*SSUrRNA*) genes had a deletion of 2 nt (G-G) and a substitution of 1 nt (C to T), when compared with the classic type, the Nigerian I/CDC strain (6). These polymorphisms had practical implications, since they occurred in the target of a diagnostic oligonucleotide probe used by the commercially available microtiter-plate hybridization (MPH) method for malaria diagnosis (4). Soon after, the same sequence variation was reported in three cases imported from Africa into Japan (7); all patients had single infections with the variant *P. ovale*. Later, variant-type sequences were found in the Cameroon (8) and MAL/MAI isolates (L.K. Basco, unpub. data), as well as in isolates from other Southeast Asian countries such as Thailand, Laos, Myanmar, and Indonesia (9–12). Four features of sequence variation in *P. ovale* soon became clear: 1) both classic and variant-type parasites occurred in sympatry (i.e., they co-occurred in the same disease-endemic areas); 2) parasites with variant-type sequences did not differ morphologically from classic parasites; 3) variant-type parasites were present in both Asia and Africa; and 4) parasites with variant-type sequences tended to produce higher parasitemia levels and higher proportions of single-species infection, when compared with classic *P. ovale* infections acquired in the same region (2,11).

In contrast with *P. falciparum* and *P. vivax*, little is known about the patterns of genetic diversity in field isolates of *P. ovale*. So far, full sequences of the *SSUrRNA* gene have been analyzed for only three isolates, the Nigerian I/CDC strain (6) and two African isolates, Cameroon and MAL/MAI; partial sequences are also available only for four isolates, the London School of Hygiene and Tropical Medicine strain (LS train) and the Nigerian I/CDC strain (13), and two isolates from Papua New Guinea (14) and Ghana (C. Severini et al., unpub. data). The cysteine protease gene was sequenced only for the Nigerian I/CDC (15), whereas the Harding strain is the only source of available sequence for the cytochrome b (*cyt b*) gene (16). More recently, two types of sequences have been characterized for the ookinete surface protein genes, *Pos 25*, *Pos 28-1*, and *Pos 28-2* in *P. ovale* isolates from Thailand (17,18). They correspond to the two types of *SSUrRNA* genes, Nigerian I/CDC and LS, which suggests that two sequence types might represent distinct variants or subspecies (13,18).

We have obtained sequence data of the *SSUrRNA*, cysteine protease, ookinete surface protein, and *cyt b* genes of *P. ovale* isolates from Myanmar and Indonesia and compared our data with GenBank-available sequences. Our analyses of both nuclear and mitochondrial genes provide further support to the division of *P. ovale* into at least two types.

## Materials and Methods

### Field *P. ovale* Isolates

All isolates were obtained during our recent field surveys in Myanmar and Indonesia (2,11,12). For molecular analysis of the variant and classic types, patients with single infections were selected. The variant isolates we analyzed were ST243 (Rakhine State) and MC53 (Tanintharyi Division), both from Myanmar, and M474 (Flores Island, eastern Indonesia). The three classic isolates of M3 (Shan State), M4 (Bago Division), and T134 (Mon State) were collected from Myanmar.

### Isolation of Parasite DNA and Confirmation of *P. ovale* by Sequence Analysis

Parasite DNA templates were isolated from blood by using a DNA isolation Kit (High Pure PCR Template Preparation Kit, Boehringer Mannheim, Germany). Then the target sequences at the block 9 region used for PCR-based diagnosis were further analyzed to confirm the presence of the variant- or classic-type in *P. ovale*–positive samples. Amplified DNA products using the P1F-Up and specific reverse (PoR2) primers (Table 1) underwent direct sequencing, whereas the first PCR products were cloned into the pCR II plasmid from a TA Cloning Kit (Invitrogen, San Diego, CA). The target fragments of 12 positive clones from each sample were sequenced by using Big Dye Terminator sequencing kit on an ABI 310 sequencer (PE Applied Biosystems, Foster City, CA.

**Table 1:** Oligonucleotide primers used in this study

Target gene	Primers	Sequences (5´→3´)
A type of the *SSUrRNA* gene	18S F	AACCTGGTTGATCTTGCCAGTAGTC
	18S F1	CGATTCGGAGAGGGAGCCTGA
	PoR2	TGAAGGAAGCAATCTAAGAAATTT
	P1F-UP	TCCATTAATCAAGAACGAAAGTTAAG
	18S F2	TGGATGGTGATGCATGGCCGT
	18S R	TAATGATCCTTCCGCAGGTTCACC
Cytochrome b gene	Cyt b 1F	ATGAATTATTATTCTATTAATTTAG
	Cyt b 1R	GGATCACTTACAGTATATCCTCC
	Cyt b 2F	CAAATGAGTTATTGGGGTGCAAC
	Cyt b 2R	TTTTAACATTGCATAAAATGGTA
	Cyt b 3F	CCAAATCTATTAAGTCTTGATGT
	Cyt b 3R	TGTTTGCTTGGGAGCTGTAATCA
Cysteine protease gene	CysP-F	GCCAGTGTAGGTAATATTGAAT
	CysP-R	GTATAAAATATCATCATCATCA
Ookinete surface protein genes		
First polymerase chain reaction (PCR)		
*Pos 25*	Po8F2	CTTTTGTTAGTATTTCCTCC
	Po8R1C	ACATTGAACACAGAATATGC
*Pos 28-1*	Po1F1	TCCCCTTTTGTCCGTTTGTC
	Po1R1	AAAGACTGCTACACGCATAC
*Pos 28-2*	Po4NF1	GTTCATTACATTAAGTTCTC
	Po4R1	TTAAATTGTATAAATTACACTG
Nested PCR		
*Pos 25*	Po8F1-in	TTACAGTTTGTTTCTCGTC
	Po8R1-in	AGGTTTAAGACATTGAACAC
*Pos 28-1*	Po1F1-in	TTTTCTTTTCGTTTGCTTGC
	Po1R1-in	TCAATATGGACACAGAATGC
*Pos 28-2*	Po4F1-in	TTTACCATTTTCCAATATGC
	Po4R1-in	CAATTAAAATTAAAATTCTG

### Analysis of *SSUrRNA*, Cysteine Protease, and *cyt b* Genes

Primer sets used were shown in Table 1. For analysis of the *SSUrRNA* gene, PCR amplification was performed by using AmpliTaq Gold polymerase (PE Applied Biosystems) at 96°C for 10 min, 36 cycles at 94°C for 30 sec, 55°C for 1 min, and 72°C for 2 min, followed by one cycle at 72°C for 10 min.

For analysis of the cysteine protease and *cyt b* genes, PCR conditions were slightly modified from the original methods (15,16). The conditions used were one cycle at 96°C for 10 min, 36 cycles at 94°C for 30 s, 50°C for 1 min, and 72°C for 90 s, followed by one cycle at 72°C for 10 min. The amplified PCR products were cloned into the pCR II vector. Plasmid DNA was purified from the positive colonies and sequenced in both directions by using the primers described in Table 1 in combination with M13 primers. Sequencing was performed with an ABI 377 sequencer. Any ambiguity and putative polymorphism was checked by additional amplification and sequencing. Sequences obtained were compared with those reported in databases.

Gene sequences used for the *SSUrRNA* genes were clone 9 and 26 of the Nigerian I/CDC strain (6), isolates from Cameroon (8), MAL/MAI (X99790), Papua New Guinea (14), Ghana (AJ250701), and two strains of the Nigerian I/CDC and the London School of Hygiene and Tropical Medicine (13). For the cysteine protease, *P. ovale* Nigerian I/CDC, *P. malariae* WR314, *P. cynomolgi*, *P. reichenowi* (15), *P. vivax* Salvador-1 (19), and *P. falciparum* (20) were used. For the *cyt b*. *P. ovale* Harding strain, *P. malariae* Uganda-1, *P. falciparum* Kenya, Santa Lucia, Malaysian-4, 3D7, *P. simiovale*, *P. knowlesi*, *P. cynomolgi* (16), *P. falciparum* Malay Camp (21) and C10 (22), *P. reichenowi*, *P. falciparum* NF54, K1, T9/96, 7G8 (23), Indian isolate 317 (24), *P. vivax* Salvador-I (25), and Indian PH 10 (26) were retrieved as well as *P. berghei* (27) and *P. yoelii* (28). Dendrograms were obtained with PHYLIP (Version 3.5c, University of Washington, Seattle, WA) by using the neighbor-joining method with a Kimura’s two-parameter distance and the maximum likelihood method.

### Sequence Analysis of *Pos 25, Pos 28-1,* and *Pos 28-2* Genes

The procedures for first and nested PCR amplifications with primers (Table 1) were described previously (17,18). Nucleotide sequences were determined by direct sequencing with nested PCR products. Then, sequences obtained were compared with those reported previously (AB051631-3, AB074973-6).

## Results

### Sequence Analysis of the Full *SSUrRNA* Gene

**Figure 1:**
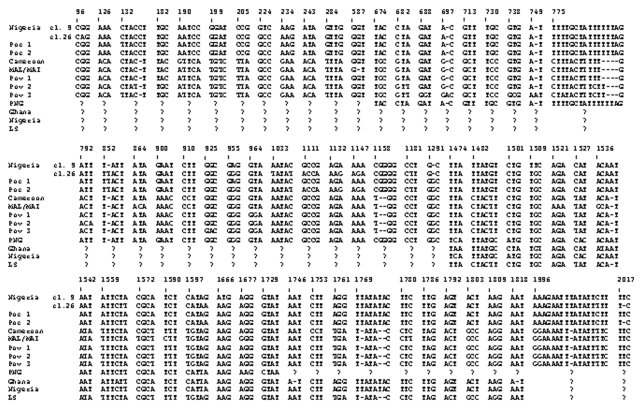
Different nucleotide sequences of the *SSUrRNA* genes among various *Plasmodium ovale* isolates. Numbers of nucleotides are based on the *P. ovale* clone 9 sequence. Boldface letters show different nucleotides in each isolate. Poc 1–2 and Pov 1–3 indicate two and three different sequences obtained from the classic and variant *P. ovale* isolates, respectively. Nigeria, Nigerian I/CDC strain; PNG, Papua New Guinean isolate; LS, a strain from the London School of Hygiene and Tropical Medicine.

Three different sequences were obtained for the *SSUrRNA* gene from each variant-type isolate, while two different sequences were detected from each classic-type isolate (Figure 1). However, whether all of them were A (asexual)-type genes or sequences included S (sexual)- or O (ookinete/oocyst)-type (5,29,30) genes was unknown. Hereafter, these sequences are referred to as Pov 1–3 for the variant-type and as Poc 1–2 for the classic-type.

The differences among Pov 1–3 were seen at the 5´ half (Figure 1). When compared with the four complete sequences in GenBank, the Cameroon and MAL/MAI isolates were grouped as variant-type (>99% identity with Pov 1–3 and <97% with Poc 1–2). Both African isolates also shared the same mutation at the block 9 region (nucleotide positions of 1158–1160). Particularly, the sequence found in Cameroon isolate resembled that of Pov 1 (only 4-bp difference).

The alignment of four partial sequences showed that, despite their same origin, the partial sequence of the Nigerian I/CDC (13) also showed 9-bp and 5-bp differences from those of clones 9 and 26, respectively. Among these isolates, the LS strain possessed the same sequence as the 3´ half of Pov 1–3, and thus it was grouped as variant type (<96% identity with Poc 1–2). The sequence of the Papua New Guinea isolate was more similar to that of clone 9 of the Nigerian I/CDC (>98.8% identity with Poc 1 >and <97% with Pov 1–3) than to that of clone 26 or Poc 2 (98.2%–98.4% identity). The Ghana isolate was also grouped as classic-type (>97% identity with the Nigerian I/CDC or Poc 1–2 and <92% with Pov 1–3). These results suggest that the Papua New Guinea, Ghana, and our classic isolates are members of the classic- (Nigerian I/CDC-) type group, whereas the Cameroon and MAL/MAI isolates, as well as our variant isolates, are all members of the variant- (LS-) type group.

### Sequence Analysis of the Cysteine Protease and the Ookinete Surface Protein Genes

The analysis of 531 bp of the cysteine protease genes, when compared with the reported sequence of the Nigerian I/CDC, showed that variant isolates differed at 19 bp (3.6%) with eight nonsilent mutations and that classic-type isolates had an almost identical sequence, except for a single base at position 700 (nonsilent substitution from Pro to Ala) (Table 2). Because the same substitution is also seen in the variant *P. ovale*, *P. falciparum*, *P. vivax*, *P. malariae*, *P. reichenowi*, and *P. cynomolgi* (data not shown), this nucleotide may have been misread in the sequence of the Nigerian I/CDC strain; if so, sequences of classic-type isolates are identical to those of the Nigerian I/CDC strain. At the amino acid level, the variant isolates showed 96.0% sequence identity with the classic isolates.

**Table 2:** *Cysteine protease* genes in different *Plasmodium ovale* isolates^a^

Position	444	471	501	552	**599**	**600**	633	**685**	**700**	720	774	786	789	**860**	**881**	**886**	**895**	**896**	**914**
Nigerian I/CDC	T	T	T	A	A	A	T	A	C	G	T	A	T	C	A	C	A	G	A
Classic isolate	T	T	T	A	A	A	T	A	G	G	T	A	T	C	A	C	A	G	A
									(P→A)										
Variant isolate	C	C	G	G	G	G	C	G	G	T	A	C	C	A	G	G	G	C	C
					(K→R)			(N→D)	(P→A)					(T→K)	(K→R)	(H→D)	(S→A)		(E→A)

^a^Nucleotide numbers in boldface indicate positions resulting in nonsilent mutations (parentheses).

Tachibana et al. (17) have analyzed the ookinete surface protein genes in Thai isolates and reported that two (Nigerian I/CDC and LS) types of *P. ovale*, defined by the *SSUrRNA* genes, have distinct sequences. Our nearly complete sequences of *Pos 25*, *Pos 28-1*, and *Pos 28-2* in variant isolates were identical to those found in LS-type Thai isolates, while sequences in the classic isolates were identical to those of the Nigerian I/CDC-type (data not shown).

### Sequence Analysis of the *cyt b* Gene

**Figure 2:**
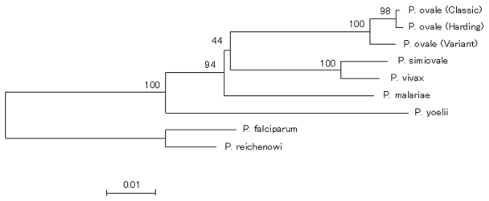
A dendrogram based on cytochrome b sequences of *Plasmodium* species including *P. ovale* variant and classic isolates. Bootstrap values are provided as percentages over 1,000 replications.

The analysis of 1035 bp of the *cyt b* genes showed that variant- and classic-type *P. ovale* isolates differed from each other at 12 bp, with one nonsilent substitution (Table 3). Sequences of variant isolates differed from those reported for the Harding strain at 15 bp (1.4%), with two nonsilent substitutions. The *SSUrRNA* gene of the Harding strain was not reported yet, and whether this strain is of the classic or variant type is not known. From its *cyt b* sequence, it was expected that this strain may belong to the classic group, despite the differences between the respective sequences (3 bp, including one nonsynonymous replacement). The dendrogram based on the *cyt b* genes shown in Figure 2 suggests that *P. ovale* may be separated into three types. A similar branching pattern was obtained with the maximum likelihood method (data not shown). However, some sequence mistakes cannot be ruled out in GenBank-available sequences, such as that of Harding strain (for example, nt 202–221 are conserved in all reported *Plasmodium* spp. so far studied, except for *P. ovale* Harding strain and *P. malariae* Uganda-1). As a result, it seems more prudent to propose the separation of *P. ovale* into only two types.

**Table 3:** *cyt b* genes in different *Plasmodium ovale* isolates^a^

Position	9	12	87	126	**211**	300	327	417	459	**669**	681	699	810	828	873
Harding strain	C	G	A	A	A	T	C	T	C	G	C	T	C	C	T
Classic isolate	T	A	A	A	G	T	C	T	C	G	C	T	C	C	T
					(R→G)										
Variant isolate	T	A	C	G	G	A	T	A	T	T	T	A	T	T	A
					(R→G)					(M→I)					

^a^Nucleotide numbers in boldface indicate positions resulting in nonsilent mutations (parentheses).

## Discussion

By analyzing the 3´ half of the *P. ovale* *SSUrRNA* genes, Li et al. (13) suggested that *P. ovale* might be separated into two types or subspecies, Nigerian I/CDC and LS. Later, the presence of LS-type or variant-type *P. ovale* was confirmed in Vietnam (4) and Africa (7); all variant-type isolates shared the same mutations at the block 9 in the *SSUrRNA* gene. Sequence analyses of the ookinete surface antigen gene, presented here and elsewhere (18), and of the cysteine protease gene all confirmed the occurrence of two different sequences in nuclear genes of parasites grouped as variant type and Nigerian I/CDC or classic type based on their *SSUrRNA* gene sequence.

Whether the different sequences of *SSUrRNA* genes we describe for classic-type and variant-type *P. ovale* isolates correspond to A genes or include S or O genes is unclear (5,29). In *P. falciparum* (30) and *P. vivax* (5), extensive pairwise sequence diversity (>13% difference) has been reported between A and S or O genes. In both classic type and the variant isolates, however, *SSUrRNA* gene sequences were quite similar to each other (<4% difference), which suggests that they may all correspond to A genes. The occurrence of different A gene–like sequences may be a distinctive feature of *P. ovale*, indicating a possible field for future research.

Because of the strict sequence conservation of the mitochondrial *cyt b* gene in natural isolates of the human malaria parasites *P. falciparum* and *P. vivax*, the divergence we found between sequences from variant- and classic-type parasites are putatively of major importance in defining two genetically distinct types of *P. ovale*. Analyzing the *SSUrRNA* gene of the Harding strain and determining whether this strain belongs to the variant-type or classic-type group or a third, poorly characterized group would be of interest.

The prevalence and geographic distribution of *P. ovale*, the last human malaria parasite to be described, have elicited little interest until recently. We have previously shown that *P. ovale* is a widespread human pathogen in Southeast Asia (1,2); here we suggest that, in both Southeast Asia and Africa, at least two different types of *P. ovale* circulate in human hosts. This situation is reminiscent of that recently described for *P. vivax*, which may be divided into two types occurring respectively in the Old World and the New World (31). However, both variants of *P. ovale* (in contrast to those of *P. vivax*) occur in sympatry, which suggests that the genetic differentiation between them is not associated with geographic isolation. Moreover, the fact that human infections with variant-type *P. ovale* tend to be associated with higher levels of parasitemia, when compared with levels associated with classic-type parasites (2,4,11), may be the result of more dramatic biologic differences between these types, with possible clinical implications.
